# Factors Associated with Haemoglobin Concentration among Timor-Leste Children Aged 6–59 Months

**Published:** 2008-06

**Authors:** K.E. Agho, M.J. Dibley, C. D'Este, R. Gibberd

**Affiliations:** ^1^School of Medicine, University of Western Sydney, NSW, Australia; ^2^School of Public Health and George Institute for International Health, University of Sydney, NSW, Australia; ^3^Centre for Clinical Epidemiology and Biostatistics, University of Newcastle, NSW, Australia

**Keywords:** Anaemia, Anaemia, Iron-deficiency, Haemoglobin concentration, Child, Preschool, Hierarchical multiple regression, Timor-Leste

## Abstract

The study was conducted to assess the prevalence of and factors associated with haemoglobin (Hb) concentrations among children aged 6–59 months in Timor-Leste. The 2003 Demographic and Health Survey was a multi-stage cluster survey of 4,320 households from four different geographic regions in Timor-Leste. In total, 4,514 children aged 6–59 months were included in the analysis. The prevalence of anaemia (Hb concentration <11.0g/dL) was 38.2% (638/1,668) for children aged 6–23 months and 22.6% (644/2,846) for older children (p<0.001). Girls had a higher mean Hb concentration than boys (11.9g/dL vs 11.7g/dL, p<0.006) and children who had diarrhoea in the previous two weeks had a lower Hb concentration than children without diarrhoea (11.5g/dL vs 11.9g/dL, p<0.001). Children from the richest and middle-class households had a lower average Hb concentration than those from the poorest households (11.8g/dL, 11.7g/dL vs 12.0g/dL, p<0.001). Children of mothers with some secondary or more education had a lower mean Hb concentration than children of mothers with completed primary, some primary and no education (11.7 g/dL vs 11.9 g/dL, 11.8 g/dL, and 11.9 g/dL, p=0.002). Children from severely-anaemic mothers had a lower mean Hb concentration than children from moderately-, mild and not anaemic mothers (10.5 g/dL vs 11.1 g/dL, 11.6 g/dL, 12.0 g/dL, p<0.001). After backward stepwise hierarchical multiple regression, wasting, male sex, recent diarrhoea, household wealth index (richest and middle-class), maternal educational status (some secondary or more and some primary), and maternal anaemic status were significantly associated with a lower Hb concentration in children and increased age of child and duration of breastfeeding (6 months) with a higher Hb concentration. Anaemia-prevention programmes among children in Timor-Leste should focus on those children aged less than two years, children with recent diarrhoea, wasted children, high socioeconomic status, and anaemic mothers.

## INTRODUCTION

Iron deficiency and anaemia continue to be two important public-health problems in developing counties. An estimated more than two billion people suffer from iron-deficiency anaemia worldwide (1). Young children and pregnant women are most affected around the world (2,3). Anaemia is defined as a low haemoglobin (Hb) concentration, and the cut-off value of 110 g/L for children aged 6–59 months usually applied (4) in settings where malaria is endemic ranges from 49% to 76% (3,5-6). Anaemia has been shown to affect cognitive development, shorten attention span, and cause irritability, fatigue, difficulty with concentration, lethargy, increased mortality, and susceptibility to infection. Consequently, anaemic children tend to perform poorly on vocabulary, reading and other tests (7). However, with appropriate preventive programmes, many cases of anaemia, including iron-deficiency anaemia, can be prevented in children.

Associated factors for Hb concentration among children aged 6–59 months in past studies included iron supplementations, home-prepared vegetable puree meal, intake of iron from meat, sex differences, age of child, bioavailability of iron in the diet, serum retinol concentration, diarrhoea, supply of clean water, environmental sanitation, and low birthweight (3,8-12).

The Timor-Leste Demographic and Health Survey (2003) report indicated that the overall prevalence of anaemia among Timor-Leste children aged less than five years (adjusted Hb less than 110 g/L) was 31.5%, the prevalence of moderate anaemia (adjusted Hb <90 g/L) was 6.1%, the prevalence of severe anaemia (adjusted Hb<70 g/L) was 0.8%, and children from anaemic mothers were more at risk (13,14).

Adequate planning of child health and nutrition programmes in Timor-Leste requires the availabili-ty of recent data from population-based surveys, including information on growth and other nutritional deficiencies. Thus, knowledge about factors associated with Hb concentration in children is essential for building effective prevention programmes. Identification of risk factors would assist targeting children who are at a heightened risk of low Hb concentration. However, no studies have been reported from Timor-Leste that analyzed factors associated with Hb concentration among children aged 6–59 months.

This study was undertaken to assess the prevalence of anaemia and to investigate factors associated with haemoglobin concentration in children aged 6–59 months living in Timor-Leste.

## MATERIALS AND METHODS

### Study design

Data for the present study were obtained from the demographic and health survey undertaken during April-August 2003 in a representative sample of households in Timor-Leste. A multistage cluster-sampling technique was used for selecting the study sample in which the entire country was stratified into four strata, i.e. Urban, Rural West, Rural Central, and Rural East.

### Selection of subjects

Sampling was carried out in three stages. In the first stage, from 498 villages called *sucos* (clusters) (13), 40 *sucos* were randomly selected based on probabili-ty proportional to size (PPS) in each stratum. In the second stage, three wards (sub-villages)—called *aldeias*—were selected with PPS from each of the 40 *sucos*. Finally, 10 households in each selected *aldeias* were visited. Each household was visited individually, and the survey included all children aged less than five years. The sample size was adapted from the Timor-Leste 2002 Multiple Indicators Country Survey sampling strategy to provide adequate sample size for the entire Timor-Leste population (13).

### Socioeconomic, demographic, breastfeeding and child morbiditycharacteristics

A structured household questionnaire was used for collecting information about the following: region of residence (Urban, Rural East, Rural West, and Rural Central); ecological zones (defined by the attitude above sea level, with highland households located at more than 1,000 metres above the sea level and lowland households located below 1,000 metres below sea level); household's ownership of consumer items (e.g. television, car; flooring material; type of drinking-water source; toilet facilities; and other characteristics that are related to wealth status); maternal education; maternal age; paid maternal work; and antenatal visits. Weight and height of mothers were measured. For the index children, the variables were age, sex, duration of breastfeeding, diarrhoea, and cough in the last two weeks collected using the standard demographic and health survey mother questionnaires.

### Biomarker measurement

The household questionnaire also included several biomarker measurements. We measured the height, weight, and haemoglobin for all children and mothers aged 15–49 years. Levels of haemoglobin were measured in the field using a portable HaemoCue instrument that provided results in less than a minute. The instrument was calibrated by a phlebotomist at the start of each day following the quality-control instructions of the manufacturer. Several studies have established the validity of HaemoCue measurements of Hb in field settings (15-17). However, in a malaria-endemic setting, one study reported a consistent bias in Hb values in children aged 6–15 months when using the HaemoCue method (18).

### Child anthropometric indicators

Three standard indices of the nutritional status of children are: height-for-age (stunting), weight-for-height (wasting), and weight-for-age (underweight).

Each of the three child nutritional status measurements was expressed in standard deviation (SD) units (z-scores) from the median of the reference population. Children with a measurement of <-2 SD from the median of the reference population are considered to be chronically (HAZ) or currently (WHZ) malnourished while children with measurement of <-3 SD from the median of the reference population are considered to be severely affected (19).

### Ethical permission

This study had the approval of the Ministry of Health in Timor-Leste and the protocol was approved by the Human Ethics Research Committee, University of Newcastle. Severely-anaemic adults and children were referred to local public-health services for treatment.

### Statistical analysis

The questionnaires were checked daily for accuracy, consistency, and completeness. Double entry was conducted by two data clerks independently and verified using the Census and Survey Processing System (CsPro). The century month codes (CMC)—methods adopted in most demographic and health survey studies—were used for determining the age of children in months, and children aged less than six months were dropped from the analysis. National and regional distribution curves were constructed for Hb concentration for children aged 6–59 months. A wealth index was constructed from data collected in the household questionnaire, using methods recommended by the World Bank Poverty Network and United Nations Children's Fund (20). The wealth index was divided into three categories. The bottom 40% of the households was referred to as the poorest households, the next 40% as the middle-class households, and the top 20% as the richest households.

Statistical analyses were undertaken with the Stata software (version 9.2) (2004; Stata Corporation, College Station, TX, USA). Exploratory data analysis was conducted using frequency distribution for categorical variables and graphs and summary statistics for continuous variables. The standard *t*-test was used for comparing the differences between two mean values. One-way ANOVA was used for more than two mean values. Multiple linear regression analysis based on the hierarchical model was used for adjusting for clustering of the primary sampling unit (PSU), geographical regions (including weights). Multiple hierarchical analyses using a backward stepwise method were used for identifying factors associated with Hb concentration in children aged 6–59 months. We considered p≤0.05 as statistically significant. All statistical tests were two-tailed.

## RESULTS

**Fig. 1:**
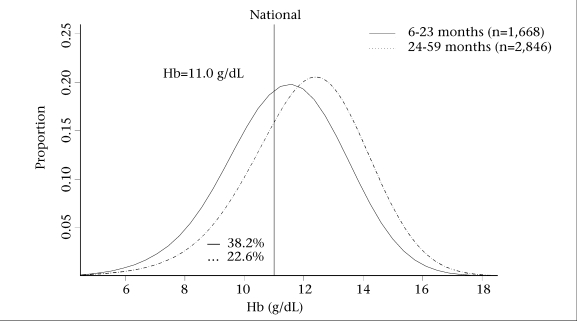
National distribution of Hb level for children aged 6–59 months in Timor-Leste

Figure 1 shows the national distribution of Hb concentration for younger children aged 6–23 months and older children aged 24–59 months (the vertical line indicates cut-off Hb concentration for diagnosis of anaemia, 11.0 g/dL). In Timor-Leste, about 38.2% of the children aged 6–23 months were anaemic compared to 22.2% of the older children aged 24–59 months.

**Fig. 2:**
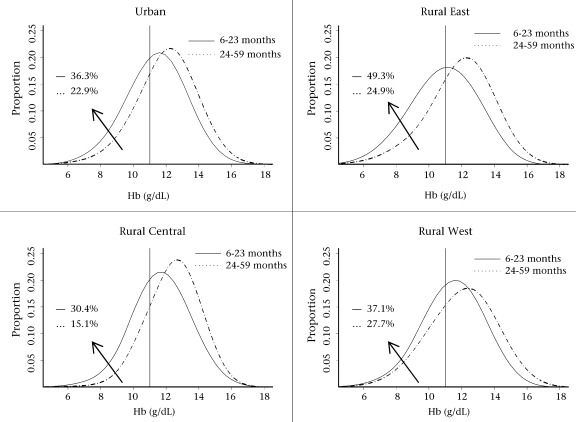
Regional distribution of Hb level for children aged 6–59 months

Figure 2 shows the diagnosis of anaemia with 11.0 g/dL in different geographical regions. In the four geographical regions, the prevalence was lower in Rural Central (30.4%) than in other three regions (Urban, Rural East, and Rural West: 36.3%, 49.3%, and 37.1% respectively). The regional average Hb levels for children aged 6–23 months were 11.4 g/dL, 10.9 g/dL, 11.7 g/dL, and 11.5 g/dL while those of older children were 12.0 g/dL, 11.9 g/dL, 12.1 g/dL, and 12.4 g/dL for Urban, Rural East, Rural Central, and Rural West respectively.

Bivariate analyses for Hb concentration by infant/child characteristics are shown in Table 1. Among the variables examined, the mean Hb concentrations of children aged 6–23 months were significantly lower than those of older children aged 24–59 months (11.4 g/dL vs 12.1 g/dL, p<0.001). The wasted children had a significantly lower mean Hb concentration than children with adequate weight-for-height children (11.7g/dL vs 11.9 g/dL, p=0.025).

**Table 1:** Bivariate analyses of age of child, nutritional status, morbidity, and Hb concentrations (g/dL) (mean values and 95% CI)

Variable	No.	Hb (g/dL)	95% CI	Statistical significance of effect
Age-group (months)				
6–23	1,668	11.4	11.3, 11.4	t_(4514)_ −13.97, p<0.001
24–59	2,846	12.1	12.0, 12.2	
Sex				
Boy	2,252	11.7	11.7, 11.8	t_(4514)_ −2.74, p=0.006
Girl	2,262	11.9	11.8, 12.0	
Height-for-age (z-score <−2 SD)				
Stunted	2,512	11.8	11.8, 11.9	t_(4259)_ −0.032, p=0.9747
Not stunted	1,749	11.8	11.7, 11.9	
Weight-for-age (z-score <−2 SD)				
Underweight	1,823	11.8	11.7, 11.9	t_(4259)_ 1.64, p=0.10
Adequate weight	2,438	11.9	11.8, 11.9	
Weight-for-height (z-score <−2 SD)				
Wasted	673	11.7	11.6, 11.8	t_(4253)_ 2.24, p=0.025
Adequate weight-for-height	3,582	11.9	11.8, 11.9	
Diarrhoea in the last 2 weeks				
Yes	450	11.5	11.4, 11.7	t_(4266)_ −3.58, p<0.001
No	3,816	11.9	11.8, 11.9	
Cough in the last 2 week				
Yes	1,404	11.8	11.7, 11.9	t_(4269)_ −0.88, p= 0.377
No	2,865	11.8	11.8, 11.9	
Duration (months) of breastfeeding				
6	146	11.9	11.6, 12.2	t_(4514)_ 0.77, p=0.439
>6	4,368	11.8	11.8, 11.9	

CI=Confidence interval; SD=Standard deviation

The girls had a significantly higher mean Hb concentration than boys (11.9 g/dL vs 11.7 g/dL, p=0.006). Children with diarrhoea in the last weeks before survey had significantly lower mean Hb concentrations than children without diarrhoea (11.5 g/dL vs 11.9 g/dL, p<0.001).

Table 2 shows Hb concentrations by demographic, socioeconomic and maternal characteristics. The mean Hb concentration was significantly lower in Rural East than in the other three geographical regions (11.5 g/dL vs Urban, Rural West, and Rural Central: 11.8 g/dL, 11.8 g/dL, and 12.1 g/dL respectively, p<0.001). The lowland households had a significantly lower average Hb concentration than that of the highland households (11.8 g/dL vs 12.0 g/dL, p<0.001). The poorest households had a significantly higher mean Hb concentration than that of the middle and richest households (11.9 g/dL vs 11.6 g/dL and 11.7 g/dL, p<0.001).

**Table 2:** Bivariate analyses of socioeconomic and maternal variables and Hb concentrations (g/dL) in children (mean values and 95% CI)

Variable	No.	Hb (g/dL)	95% CI	Statistical significance of effect
Region				
Urban	1,179	11.8	11.7, 11.9	F_(3,4510)_ 27.86, p<0.001
Rural West	1,126	11.8	11.7, 11.9	
Rural Central	1,113	12.1	12.1, 12.2	
Rural East	1,096	11.5	11.4, 11.6	
Ecological zone				
Lowlands	3,891	11.8	11.7, 11.8	t_(4514)_ −3.49, p < 0.001
Highlands	623	12.0	11.9, 12.2	
Household wealth index				
Poorest	1,876	12.0	11.9, 12.1	F_(2,4511)_ 19.47, p<0.001
Middle	1,779	11.7	11.6, 11.8	
Richest	859	11.8	11.7, 11.9	
Latrine				
Yes	1,305	11.8	11.7, 11.9	t_(4514)_ −0.33, p=0.742
No	3,209	11.8	11.8, 11.9	
Maternal education				
No education	2,028	11.9	11.8, 12.0	
Some primary	840	11.8	11.7, 11.9	F_(3,4391)_ 4.90, p=0.002
Completed primary	377	11.9	11.7, 12.0	
Some secondary or more	1,150	11.7	11.6, 11.8	
Age (years) of mothers at birth				
15–19	69	11.3	10.9, 11.7	F_(3,4476)_ 4.05, p=0.007
20–24	617	11.9	11.8, 12.1	
25–29	1,128	11.8	11.7, 11.9	
30–49	2,666	11.9	11.8, 11.9	
Maternal working status				
Working	1,788	11.8	11.7, 11.9	t_(4514)_ −0.01, p=0.996
Non-working	2,726	11.8	11.8, 11.9	
Maternal antenatal visit				
Once	123	11.6	11.3, 11.9	F_(2,4511)_ 6.38, p=0.002
More than once	1,465	11.7	11.6, 11.8	
None	2,926	11.9	11.9, 12.0	
Maternal body mass index				
≤18.5	1,489	11.7	11.6, 11.8	t_(4367)_ −2.62, p=0.008
>18.5	2,878	11.9	11.8, 11.9	
Maternal anaemic status				
Not anaemic	2,903	12.0	12.0, 12.1	F_(3,4413)_ 45.33, p<0.001
Mild anaemic	1,242	11.6	11.5, 11.7	
Moderate anaemic	224	11.1	10.9, 11.4	
Severe anaemic	48	10.5	10.0, 11.0	

CI=Confidence interval

Children of mothers with some secondary education had a significantly lower mean Hb concentration than children of mothers with completed primary, some primary and no education (11.7 g/dL vs 11.9 g/dL, 11.8 g/dL, and 11.9 g/dL, p=0.002). Children of younger mothers had a significantly lower mean Hb concentration than children from older mothers (11.3 g/dL, vs 11.9 g/dL, 11.7 g/dL, and 11.8 g/dL, p=0.007). Children of severely-anaemic mothers had a significantly lower mean Hb concentration than children from moderately-, mildly- and not anaemic mothers (10.5 g/dL vs 11.1 g/dL, 11.6 g/dL, 12.0 g/dL, p<0.001). Mothers with low body mass index (BMI) had a significantly lower mean Hb concentration than children of mothers with adequate BMI (11.7 g/dL vs 11.9 g/dL, p=0.008).

### Hierarchical multiple regression

Hierarchical multiple regression analyses were performed for the outcome variable. Results of the hierarchical multiple regression analysis using backward stepwise methods are shown in Table 3. The results revealed that increased age of children in months significantly increased Hb concentration in children by an average of 0.03 per month (confidence interval [CI] 0.02–0.03, p<0.001) which implies an increase of one year in age, the Hb increases by 0.36. In wasted children, Hb concentration was significantly reduced by an average of 0.21 (CI −0.33 to −0.09, p=0.001). Hb concentration in boys was significantly lower than girls by a mean of 0.10 (CI −0.20 to −0.01, p=0.036). The duration of breastfeeding for six months increased Hb concentration in children by a mean of about 0.48 (CI 0.88–0.87, p=0.017). In children with diarrhoea, Hb concentration was significantly reduced by an average of 0.21 (CI −0.38 to −0.03, p=0.019).

**Table 3:** Factors associated with Hb concentrations (g/dL) in Timor-Leste

Factor	Coefficient	95% Cl	p value
Age (months) of child	0.03	0.02, 0.03	<0.001
Weight-for-height (z-score < −2 SD)			
Wasting	−0.21	−0.33, −0.09	0.001
Sex			
Boy	−0.10	−0.20, −0.01	0.036
Duration (months) of breastfeeding			
6	0.48	0.088, 0.87	0.017
Diarrhoea in the last 2 weeks			
Yes	−0.21	−0.38, −0.03	0.019
Household wealth index			
Middle	−0.30	−0.44, −0.15	<0.001
Richest	−0.19	−0.40, 0.02	0.074
Maternal educational status			
Some primary	−0.01	−0.14, 0.12	0.883
Completed primary	0.01	−0.20, 0.22	0.930
Some secondary or more	−0.16	−0.31, −0.01	0.033
Maternal anaemic status			
Mild anaemic	−0.39	−0.52, −0.26	<0.001
Moderate anaemic	−0.79	−1.11, −0.47	<0.001
Severe anaemic	−1.26	−1.75, −0.77	<0.001
Constant	11.54	11.36, 11.73	0.000

CI=Confidence interval; SD=Standard deviation

Compared to children of the poorest households, children belonging to the richest households had a lower mean Hb concentration of 0.19 (CI −0.40 to 0.02, p=0.074) while children from the middle-class households had a significantly lower mean Hb concentration of 0.30 (CI −0.44 to −0.15, p<0.001). Children of mothers with some secondary education were likely to have a significantly lower mean Hb concentration (coefficient=−0.16, CI −0.31 to −0.01, p=0.033) than children of mothers with no education while children of mothers with completed and some primary education (coefficient=0.01, CI −0.20 to 0.22, p=0.930 and coefficient=−0.01, CI −0.14 to 0.12, p=0.883 respectively) were not signifi-cantly associated with Hb concentration in mothers with no education.

Children of severely-, moderately- and mildly-anaemic mothers were significantly associated with a lower mean Hb concentration than not anaemic mothers (coefficient=−1.26, CI −1.75 to −0.75, p<0.001), (coefficient=−0.79 CI −1.11 to −0.47, p<0.001) and (coefficient=−0.39, CI −0.52 to −0.26, p<0.001 respectively).

The final model after backward stepwise hierarchical multiple regression explained about 11.3% of the variance of Hb concentration in children and the *R*^2^ for regression produce was significant [F (13,159)=31.27, p<0.001].

## DISCUSSION

The purpose of this study was to assess the prevalence of, and the factors associated with, Hb concentration in a representative sample of households of all eligible children aged 6–59 months in Timor-Leste. This survey provides the first national representative data on Hb concentrations in Timor-Leste. The sampling method, appropriate adjustment for sampling design, including sampling weight, and a very high response rate (98%) to the survey interview are important strengths of the survey.

The study revealed the high prevalence of anaemia—31.3% in children aged 6–59 months, with the highest rate of 38.2% in children aged 6–23 months. The study also reported that children aged 6–23 months had an average of 7.4 g/dL Hb concentration, which is lower than older children. Although the households raised small animals, such as pigs, chickens, goats, sheep, and vegetables in garden as part of household foods, these were not for consumption rather for sale, and parents were not aware of the relevant nutritional value of animals protein and other sources of vitamin A for children aged 6–23 months.

A decline in Hb concentration during the first two months of life in Timor-Leste may be attributed to the years of unrest and instability in the country prior to the official independence in September 2002 and the high fertility rate (13). Previous studies have found parity to be an important factor in iron deficiency in pregnant women (21). In Timor-Leste, where the fertility rate is high (about 7.8 babies per woman), closely-spaced pregnancies may reduce iron stored in the women's body. However, the present study noted a considerable difference in Hb concentrations among children aged 6–23 and 24–59 months (Fig. 1) in four regions, and the distribution plot (Fig. 2) showed that the national prevalence of low Hb concentration among children aged 6–23 months was very high compared to that for older children. The low prevalence of anaemia among children aged 6–23 months in Rural Central is likely due to the effect of high altitude, and the prevalence of malaria is low at this altitude because transmission is less frequent.

Bivariate analyses revealed that age, gender, weight-for-height, recent diarrhoea, region, ecological zone, wealth index of households, status of maternal education, maternal age at birth, maternal antenatal visit, maternal BMI, and maternal anaemic status were significantly associated with Hb concentrations.

Children of younger mothers aged less than 20 years were more likely to have low Hb concentrations (Table 3). This is likely due to the inability of mothers to meet the high nutritional demands for adolescent growth (4). Hb concentration was higher in girls than in boys, a result similar to the finding reported by a study in South-East Asia (11).

Hierarchical regression analysis revealed that the following factors were significantly associated with higher and lower Hb concentrations in children after backward stepwise method: (a) maternal education (being some secondary); (b) wealth index of households (middle-class); (c) maternal anaemia (mild, moderately, and severe); (d) recent diarrhoea; (e) wasted (z-score <-2 SD); (f) sex (being a boy) were significantly associated with lower mean Hb concentrations while increased age of child and duration of breastfeeding (6 months) had significantly higher Hb concentrations in children. This pattern supports the findings from Brazil where breastfeeding practices until 12 months of life were significantly associated with the highest levels of Hb (22), and Senarath *et al*. argued that the breastfeeding practices in Timor-Leste were satisfactory (23).

Diarrhoea in the last two weeks remained in the final model after backward stepwise method. Diarrhoea in childhood was mainly watery, and not much blood loss was observed. Further, recent diarrhoea was unlikely quickly to affect Hb levels without acute blood loss. Therefore, the effect of recent diarrhoea was more likely to be explained by reduced feeding and decreased absorption of iron and protein during diarrhoea, with recent diarrhoea being a marker for chronic or recurrent diarrhoea.

Wasting had a significant and independent effect on Hb concentration in children aged 6–59 months. The differences in wasting mean Hb concentration of 11.7 and 11.9 translated to a change in the prevalence of anaemia of 29.6% vs 33.0% for adequate weight-for-height and wasting respectively. Wasting is often associated with acute starvation or severe disease (24). Maternal BMI had a significant and independent effect on Hb concentration in children aged 6–59 months after controlling for other variables. This finding demonstrates the importance of maternal Hb status in protecting low-birthweight newborns from anaemia. It is possible that maternal anaemia is directly and causally related to anaemia in the infant, which may persist in the child (5,8,25).

Children of the richest and middle-class households had lower mean Hb concentrations than those of the poorest households. The significant relationship between the poorest households and higher Hb mean concentration in Timor-Leste children are likely to due to geographical zones. Households with the lowest socioeconomic status were from Rural Central while those households which were from the richest and middle-class were mostly from the low land urban region where malaria is common. The significant relationship between socioeconomically-privileged groups and the lower Hb concentration in children may be as a result of the differences in wealth of households in Timor-Leste which are relatively small due to poverty. However, these findings contradict the findings of Muniz *et al.* who argued that anaemia in the western Brazilian Amazon was significantly more prevalent among children in the lowest socioeconomic stratum (26).

The study also revealed that children of some secondary-educated mothers were likely to have lower average Hb concentrations than children of mothers with no education. This may be due to poor breastfeeding practices among socioeconomi-cally-privileged groups in Timor-Leste (23,27). In Indonesia, maternal education and household economic status have continued to be very strong predictors of the nutritional outcomes of children (28).

Some limitations that need to be considered when interpreting the results of this study include: (a) no firm conclusions can be made on the causes and effect in a cross-sectional design; (b) also, the cross-sectional design did not allow the exact determination of effects of socioeconomic factors on children's Hb concentration; and (c) a number of confounding variables, such as child weight at birth, iron intake by mothers and children that may influence Hb concentration in children were not included in our analysis. The results from the backward stepwise hierarchical multiple regression model showed small *R*^2^ values. The reasons for this might be that the increase of Hb concentration is not linear, and the data entered into the backward stepwise model as risk factors were not well-categorized (3,29).

The results of the present study provided baseline information on socioeconomics, demographic and other factors potentially influencing anaemia of children in Timor-Leste. The results suggest that, in addition to age of the child, diarrhoea and weight-for-height (wasting) are directly associated with Hb levels. Maternal anaemic status is a marker for low Hb concentration in children aged 6–23 months. This result supports the need for health programmes aimed at children aged less than two years, children with recent diarrhoea, wasted children, privileged socioeconomic groups, and anaemic mothers.
